# Congenital Pulmonary Airway Malformation Type 1 With KRAS Mutation: A Case Report and Literature Review

**DOI:** 10.1155/crog/4960286

**Published:** 2026-08-19

**Authors:** Eleonora Nardi, Chiara De Blasi, Chiara Bartolini, Francesca Gensini, Lucia Pasquini, Francesca Castiglione

**Affiliations:** ^1^ Department of Health Sciences, Section of Pathology, University of Florence, Florence, Italy, unifi.it; ^2^ Department of Health Sciences, Section of Obstetrics and Gynecology, Careggi Hospital, University of Florence, Florence, Italy, unifi.it; ^3^ Department of Experimental and Clinical Biomedical Sciences “Mario Serio, ” Medical Genetics Unit, University of Florence, Florence, Italy, unifi.it; ^4^ Department for Women and Child Health, Fetal Medicine Unit, Careggi University Hospital, Florence, Italy, aou-careggi.toscana.it

**Keywords:** case report, congenital lung disease, congenital pulmonary airway malformation, histological examination, KRAS pathogenic variant

## Abstract

Congenital pulmonary airway malformation (CPAM) is a rare developmental anomaly of the fetal lung characterized by abnormal branching morphogenesis, resulting in cystic and/or solid masses within the pulmonary parenchyma. These cysts can range in size and symptoms: from small and asymptomatic to large and clinically evident. CPAM is typically identified during prenatal ultrasonography as an abnormal lung mass, predominantly involving a single pulmonary lobe. We herein describe a case of a fetus diagnosed with CPAMs type 1 harboring a *KRAS* pathogenic variant.

## 1. Introduction

Congenital lung malformations are rare developmental anomalies of the lung, including congenital pulmonary airway malformations (CPAMs) [1].

CPAMs are characterized by an abnormal growth and structure of lung tissue, often resulting in cystic lesions or malformed airways. They are typically unilateral and commonly involve a single pulmonary lobe, with an incidence of 1 per 4000 live births whereas cystic lung conditions in general are detected in about 1 per 35,000 live births [2].

These abnormalities arise from disruptions during lung development, particularly affecting the branching morphogenesis of the bronchioles [3]. Depending on the type and severity, CPAMs can lead to various respiratory symptoms and may require surgical intervention [4].

CPAM should be considered in the differential diagnosis of congenital lung lesions. These entities include bronchopulmonary sequestration (either intralobar or extralobar), characterized by the absence of communication with the tracheobronchial tree and receiving blood from an abnormal systemic artery. Other conditions include congenital lobar hyperinflation, caused by a defect in the cartilage of the bronchial wall; bronchogenic cyst, arising from abnormal development of the primitive foregut; and congenital bronchial atresia, which results in bronchial interruption with mucus accumulation and distal hyperinflation [1, 5, 6]. These entities may share overlapping prenatal and postnatal imaging features and are often distinguished based on vascular supply, anatomical location, echocardiographic, and histopathological characteristics [6].

Stocker originally proposed a classification system based on cyst size and histology identifying five different CPAMs subtypes [7]. This classification has been recently revised to emphasize the pathogenic mechanisms underlying these lesions. Notably, some subtypes are now regarded as distinct clinical entities rather than variants within a single malformation spectrum. So‐called “Type 0” lesions are no longer classified as CPAM but instead represent a diffuse developmental abnormality, currently termed acinar dysplasia [8].

Types 1 and 3 have been associated with somatic KRAS mutations that may confer a risk of malignant transformation, particularly to mucinous adenocarcinoma. Type 2 lesions, the predominant subtypes, are now interpreted as secondary to bronchial atresia occurring during fetal development [9].

Finally, Type 4 CPAM remains inconsistently defined in the literature. A substantial proportion of lesions previously classified within this category are now recognized as DICER1‐associated cystic pleuropulmonary blastoma, a distinction with significant diagnostic and prognostic implications [10,11].

Typically, CPAMs are diagnosed prenatally during routine ultrasonography, thus improving the immediate perinatal management [12, 13]. Instead, computed tomography (CT) can confirm the diagnosis postnatally, providing more information about size and location of the malformation [13, 14].

Once the diagnosis has been made, further evaluation is recommended, as CPAM can be associated with other congenital anomalies. Nevertheless, currently, it is recognized that the incidence of chromosomal abnormalities and genetic syndromes is not increased. Some defects in other systems can be associated, mainly cardiac, renal, and tracheoesophageal fistulas, but these are found only in 10% of cases [14, 15].

Clinical presentation is variable, ranging from a complete regression in utero during the third trimester (which is only apparent because the mass is still present, but due to the echogenicity of the normal lungs, it becomes undetectable) or causing severe mass effects resulting in life‐threatening conditions such as hydrops fetalis and fetal demise [13, 14].

Regarding delivery planning, there is no indication for cesarean section or preterm delivery when the lesion is small or appears to regress, and there are no signs of mediastinal shift or hydrops [16].

In contrast, in cases of large lesions that cause mediastinal displacement and/or hydrops, delivery should be planned in a tertiary care center with neonatal intensive care and access to pediatric surgery [16–18].

Treatment and prognosis depend on the size of the malformation and the clinical presentation [19]. Some infants may be asymptomatic, whereas others might exhibit difficulty or rapid breathing, cyanosis, and recurrent infections [1]. For asymptomatic cases, there is controversy regarding postnatal management. In fact, although a conservative option based on clinical monitoring is possible, the risk of infection and potential malignant development of the lesion should not be underestimated [6, 20]. Based on this observation, removal of the involved segment with a good long‐term survival is suggested [1].

Instead, if breathing difficulties are present, surgery is suggested to remove the affected lobe [1].

When a prenatal diagnosis is made, treatments ranging from maternal steroid administration to the insertion of a thoraco‐amniotic shunt are possible [16].

Steroids represent the only medical option and first‐line therapy before 32 weeks in hydropic fetuses affected by CPAM [21]. The therapeutic mechanism may be related to accelerated lung maturation or steroid‐induced mass involution that also results in resolution of hydrops [13, 22]. Shunting is indicated for large lung lesions and hydrops, as it reduces the mass effect of the malformation, improving hydrops and increasing fetal survival. The introduction of these therapeutic approaches has made the option of fetal surgery very rare and limited to large lesions with persistent mediastinal shift [23].

Possible postnatal complications of CPAMs include spontaneous pneumothorax, hemopneumothorax, and pyopneumothorax. The risk of malignant transformation in neonatal lesions remains uncertain [4].

The molecular pathogenesis of CPAMs involves both genetic and developmental factors [24]. Several genes implicated in lung development have been identified to contribute to these malformations [23].

We herein describe a case of a fetus diagnosed with CPAMs type 1 harboring a *KRAS* pathogenic variant.

## 2. Case Presentation

**Figure 1:**
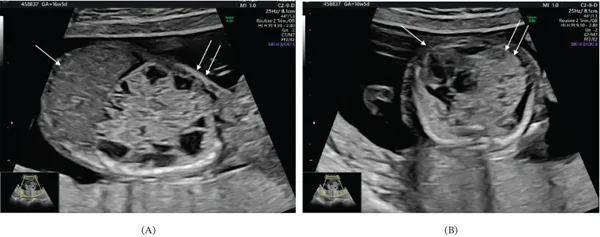
Ultrasound examination at 16 + 5 weeks. (A) A mass with mixed echogenicity and cystic appearance (double white lines). (B) The thoracic mass compressing the heart (single white line).

A 37‐year‐old woman, with two previous spontaneous miscarriages, underwent a first‐trimester ultrasound examination, which showed normal nuchal translucency. A subsequent ultrasound examination performed at 16 + 5 weeks of gestation revealed that the fetal right hemithorax was completely occupied by a mass with mixed echogenicity (23.7 mm LL × 22.5 mm AP × 33.7 mm CC), which displaced the mediastinum to the left. The heart had a normal structure but appeared displaced to the left side and compressed (Figure 1). Additional findings include ascites (4.5 mm at the level of the liver), head skin edema, and mild pleural effusion.

No other congenital anomalies were observed.

**Figure 2:**
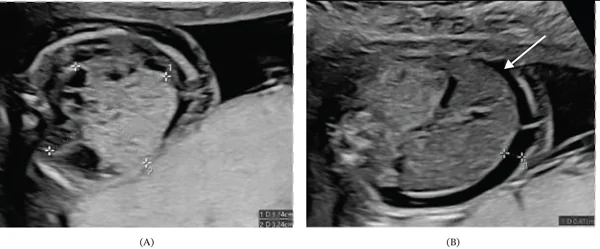
Ultrasound examination at 17 + 6 weeks. (A) An increase in the size of the mass in the right hemithorax (D1: latero‐lateral diameter; D2: anteroposterior diameter). (B) Worsening of ascites (D1) and skin edema (white line).

At 17 + 6 weeks of gestation, a follow‐up ultrasound examination was performed showing a worsening of the fetal clinical status. The right hemithorax was completely occupied by a mass with mixed echogenicity, which had increased in size (37.4 mm LL × 32.4 mm AP × 39 mm CC), with further displacement of the mediastinum to the left (Figure 2). The CVR was 0.66 at 16 + 5 weeks of gestation but had risen to 1.58 by 17 + 5 weeks of gestation, indicating a very rapid progression of the clinical course.

The mass appeared capsulated, with mixed solid‐cystic contents and macrocysts. Cardiac compression was more pronounced. Ascites had increased in volume compared with the previous ultrasound (4.7 mm at the level of the liver); head skin edema and pleural effusion had significantly worsened.

The initial ultrasound suspicion was of a possible pulmonary neoplastic mass due to the rapid and even early growth of the lesion. However, most intrauterine thoracic tumors have benign characteristics; in fact, hydrops is not related to the malignancy of the mass but rather to mediastinal compression.

The patient also underwent amniocentesis for genetic testing (CGH array and karyotype test) showing no abnormalities.

The ultrasound findings were further discussed with the parents, offering the possibility of in‐utero therapy, such as fluid aspiration, thoraco‐amniotic shunt placement, or administrations of steroids, nevertheless (Figures 1 and 2). However, they chose to terminate the pregnancy at 19 weeks of gestation.

Postmortem examination revealed a phenotypic male fetus with a globular abdomen and generalized skin edema. Macroscopic examination of the thoracic and abdominal cavities demonstrated ascites with leakage of citrine fluid (approximately 10 cc).

**Figure 3:**
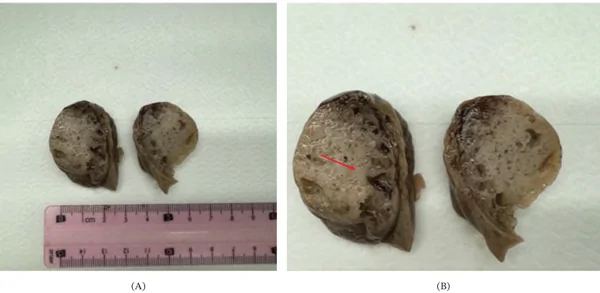
(A, B) Gross examination of the right lung: Cystic lesions can be observed; largest cyst was 22 mm in greatest dimension (red arrow in Panel (B)).

The right lung was composed of two lobes, nearly completely replaced by whitish, cystic formations. The lesions were well circumscribed and multiloculated, measuring 35 × 26 mm in diameter (Figure 3). The adjacent lung parenchyma appeared compressed but otherwise preserved. The left lung also consisted of two lobes and appeared macroscopically unremarkable. The heart appeared left dislocated.

The other organs did not show any macroscopic abnormalities.

**Figure 4:**
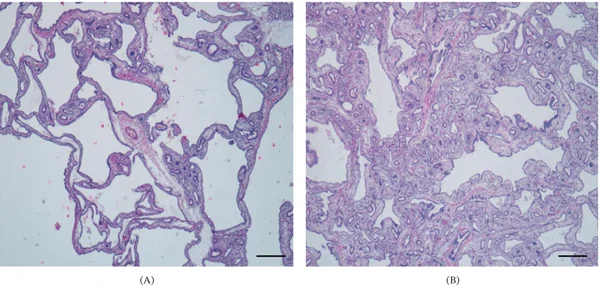
(A, B) Histologic images showing cysts lined by columnar epithelium ((A, B) H&E magnification 10x, scale bars: 100 *μ*m).

Histological examination of the cystic right lung revealed multiple cysts lined by ciliated pseudostratified columnar epithelium resembling that of normal bronchioles (Figure 4). The interstitium showed minimal chronic inflammatory infiltrate. Sampling included six paraffin blocks, providing adequate representation of the entire lesion. No evidence of dysplasia, atypical proliferation, or mucinous cell clusters was observed. In addition, the vascular supply appeared normal, with no features suggestive of sequestration.

Histological findings were compatible with CPAM.

The left lung parenchyma was normal and consistent with the reported gestational age.

Microscopic evaluation also revealed a normally developed nephrogenic cortex in the kidneys and foci of extramedullary erythropoiesis in the liver.

Placental histological examination demonstrated predominantly hydropic and avascular chorionic villi, intermixed with fibrotic villi and focal areas of immature villi. Abundant deposits of intervillar fibrin were present. The umbilical cord was composed of three vessels without significant alterations of Wharton′s jelly.

DNA was extracted from a formalin‐fixed and paraffin‐embedded (FFPE) lung tissue sample.

DNA was obtained by manually dissecting 2 × 10 *μ* *m* sections of FFPE lung tissue samples. Genomic DNA (gDNA) was extracted using “MagCore genomic DNA FFPE one‐step kit” (Diatech‐Labline Pharmacogenetics, Jesi, Ancona).

After DNA extraction, the sample was sequenced using a 16‐gene panel by means of next‐generation sequencing (Myriapod NGS‐L T 16G Oncopanel Kit, Diatech‐Labline Pharmacogenetics, Jesi, Italy) on the Ion S5 System Ion Torrent platform.

Analysis of the NGS results and identification of any genetic variants in the target regions were carried out using the “Myriapod NGS Data Analysis Software.”

Molecular analysis identified a pathogenic variant in the *KRAS* gene: c.35G > A and p.Gly12Asp (pG12D), with a variant allele frequency of 17.24%.

The histological findings together with the presence of a pathogenic *KRAS* mutation confirmed the diagnosis of CPAM type 1.

## 3. Discussion

CPAM is the most common congenital lung disorder accounting for 25% of all lung malformation [24]; it is characterized by five different subtypes based on the size and the histology of the cyst [25].

Prenatal diagnosis of CPAM is primarily based on ultrasonographic findings, typically first detected during the second trimester of pregnancy. Lesions can increase in size until 26 weeks of gestation, after which they tend to stabilize or regress. During the late second and the third trimester, spontaneous sonographic resolution (11%–49%), regression (18%–42%), or progression (33%–44%) may be observed [21, 23].

Serial prenatal ultrasound examinations are recommended every 1–4 weeks to monitor changes in size of the lung mass, changes in congenital pulmonary airway malformation volume ratio (CVR), and detect the development of polyhydramnios and hydrops. Hydrops and poor cardiac function are associated with a higher risk of perinatal death [23].

With regard to postnatal management, CPAM remains controversial, particularly in infants who are asymptomatic at birth. Nevertheless, contemporary pediatric surgical practice generally favors elective resection of most CPAMs, within the first year of life [24], because of the risks of recurrent pulmonary infections, pneumothorax, and the potential, although rare, for malignant transformation [25].

Instead, babies presenting with respiratory distress require early surgical intervention, often during the neonatal period [24].

Jelin et al. [26] demonstrated that elective resection performed between 3 and 12 months of age is safe and associated with low morbidity. Furthermore, both open and thoracoscopic resections performed at a younger age have been associated with shorter operative times.

Video‐assisted thoracoscopic surgery (VATS) has become the preferred approach for elective CPAM resection in specialized pediatric centers. Thoracoscopic lobectomy is currently considered the standard minimally invasive procedure for lesions confined to a single lobe, as it is both safe and effective. Compared with thoracotomy, VATS offers several advantages, including reduced postoperative pain, shorter hospital stay and recovery time, improved cosmetic outcomes, and a lower risk of long‐term musculoskeletal complications [27, 28].

Although conservative management is increasingly considered for carefully selected asymptomatic patients, long‐term surveillance protocols remain poorly defined. Concerns regarding infection, malignant transformation, and diagnostic uncertainty continue to support recommendations for prophylactic surgical resection in many cases. When a nonoperative strategy is chosen, postnatal cross‐sectional imaging and long‐term clinical follow‐up are recommended to monitor for respiratory symptoms, recurrent infections, lesion growth, or radiologic changes suggestive of malignancy [25].

In our case, the suspicion of a lung mass was raised at 16 weeks of gestation, highlighting the unusually early onset and rapid progression of the lesion, both of which are associated with a poorer prognosis. In fact, from the first finding, the mass almost completely occupied the right hemi thorax and the mediastinum, shifting the heart to the left. The presence of hydrops further indicated an unfavorable prognostic factor.

In addition, the extensive bilobar involvement represents another distinctive feature of our case and may be attributable to an early developmental insult occurring during branching morphogenesis [3]. However, this interpretation remains hypothetical and should be considered speculative at present. Further clinical studies and additional case observations will be necessary to better elucidate the underlying pathogenic mechanisms.

The molecular basis of CPAM, especially Type 1, involves both genetic and developmental factors [29]. Different genes (e.g. EGFR, FGFR2, and KRAS) have been identified as being involved in lung development and they contribute to CPAM [20, 30].

CPAM type 1 has also been associated with the development of mucinous adenocarcinoma.

Mucinous cell clusters are identified in approximately 70%–75% of Type 1 lesions [31]; these clusters are considered potential premalignant precursors for mucinous adenocarcinoma. Regarding malignant transformation, CPAM is associated with lung tumors in more than 20% of cases [32].

The c.35G > A mutation refers to a nucleotide change in the *KRAS* gene that results in an amino acid substitution at Position 12 of the KRAS protein (from glycine to aspartic acid).

This mutation leads to constitutive activation of the KRAS protein, causing a continuous activation of different pathways (for example MAPK/ERK and PI3K/AKT pathways) that lead to uncontrolled cell growth and proliferation [33].

Particularly, the G12D mutation is important in the context of lung cancer being the most common mutation identified in lung adenocarcinoma.

Although *KRAS* mutations have been implicated in malignant transformation in CPAM, particularly in Type 4 lesions [34], their role in Type 1 CPAM remains incompletely defined.

In particular, it should be remembered that the detection of a KRAS mutation should not be interpreted as evidence of an inevitable or clearly established risk of malignant transformation.

Nelson et al. demonstrated that CPAM type 1 results from somatic *KRAS* mutations detected in both mucinous and nonmucinous epithelial components [8].

We searched the PubMed and Embase databases for previous reports of CPAM type 1 harboring KRAS G12A mutation.

The articles found all reported babies with a diagnosis made antenatally by fetal ultrasound and subsequently managed with postnatal surgical resection. Then, the histological examination and molecular analysis were performed revealing a CPAM type 1 with *KRAS* G12A mutation. Postoperative recovery was uneventful for all of them.

The characteristics of these cases are reported in Table 1.

**Table 1:** Cases of CPAM type 1, antenatally diagnosed, reporting KRAS G12A mutation.

Reference	Gestational weeks at diagnosis	Gestational weeks at birth	In‐utero therapy	Surgery
				Type and age
Stephanov et al. ^35^	30 weeks	N/A.	N/A	15 days after birth; right lower lobe lobectomy
Koh et al. ^36^	22 + 0 weeks	39 + 2 weeks.	N/A	10 days after birth; lower lobe lobectomy
Koopman et al.^30^	20 weeks	30 + 1 weeks; spontaneous birth.	23 + 4 weeks thoraco‐amniotic shunt	6 weeks after birth; lobectomy
Muntean et al. ^37^	20 weeks	38 weeks.	21 weeks thoraco‐amniotic shunt	3 days after birth; left‐sided muscle sparing thoracotomy and lobectomy (left lung)
		C‐section.		
			N/A	8 days after birth; muscle sparing thoracotomy and lobectomy left lower lobe
	20 weeks	40 weeks; C‐section.		
Gopalaswamy et al. ^38^	2nd trimester	39 weeks; C‐section.	N/A	2nd day of life; lobectomy (right middle lobe)
Our case	16 + 5 weeks	Interruption of pregnancy.	N/A	N/A

Abbreviation: N/A, not applicable.

Two case series have been taken into account for the literature review.

Windrich et al. [39] reported a series of 43 patients with CPAM (median age of 17 months) among whom KRAS c.35G > A mutations were identified in 10 out of 23 patients with CPAM type 1.

Instead, Chang et al. [40] evaluated a cohort comprising 33 cases of CPAM type 1 and 4 cases of CPAM type 2. The patients had a mean age of 20.3 years. In this series, no cases with KRAS c.35G > *A* pathogenic variant were reported. Instead, the most frequent mutations in case of CPAM type 1 were G12V and G12D [40].

Nevertheless, this study showed that adenocarcinomas arising in CPAMs were usually indolent mucinous tumors, including in situ adenocarcinomas or invasive mucinous adenocarcinomas, and were effectively treated with anatomical resection, most commonly lobectomy.

In conclusion, we reported a case of CPAM in which the diagnosis was established thanks to the integration of prenatal imaging, histological examination, and molecular analysis, underlining the importance of a multidisciplinary approach.

Larger case series with complete ultrasound and molecular profiling could further help to clarify pathogenesis and progression of CPAM type 1 with *KRAS* pathogenic variant.

## Author Contributions

Conceptualization: E.N. and F.C.; data acquisition: E.N., C.D.B., and C.B.; writing: E.N. and C.D.B.; review: E.N., C.D.B., C.B., F.G., L.P., and F.C.

## Funding

No funding was received for this manuscript.

## Ethics Statement

The present study complied with the Ethical Principles for Medical Research Involving Human Subjects according to the World Medical Association Declaration of Helsinki; written informed consent was obtained from the patient for publication of this case report and any accompanying images. All clinical, instrumental, and histopathological data were fully anonymized prior to publication to ensure patient confidentiality.

## Conflicts of Interest

The authors declare no conflicts of interest.

## Data Availability

Research data are not shared.
